# Rezum: Analysis of the Tolerability and Complications of the Procedure Performed Under Local Anaesthetic

**DOI:** 10.3390/jcm15072560

**Published:** 2026-03-27

**Authors:** Rowan Burns, Barend Dreyer, Sinan Khadhouri, Feras Al Jaafari

**Affiliations:** 1 NHS Fife, University Health Board, Kirkcaldy KY2 5AH, UK; 2 School of Medicine, University of St Andrews, St Andrews KY16 9TF, UK

**Keywords:** Rezum, benign prostatic hyperplasia, MIST

## Abstract

**Background/Objectives:** Rezum therapy is a novel, minimally invasive way of treating benign prostatic hyperplasia (BPH) that involves the injection of heated water vapour into the prostate. It was approved by NICE in 2018 and is now available in select centres across the UK. It has been shown to have significant advantages over standard BPH therapies: it can be done under local anaesthetic, making it an option for those unsuitable for general anaesthetic, it is suitable for treating patients who want to maintain ejaculation, and it is cost-effective. It has been recommended as a treatment for smaller prostates (<80cc) and in cases where patients are keen to preserve ejaculatory function. Our unit performs this procedure under local anaesthetic (LA) with a transperineal ultrasound-guided peri-prostatic block and urethral lidocaine gel in the clinic. We aimed to analyse the patients undergoing Rezum in our institution to establish its tolerability under local anaesthetic, its effectiveness and its complication rate. **Methods:** We analysed all patients who underwent Rezum prostate steam ablation in our institution between May 2023 and September 2025. From individual patient notes, we collected data on patient demographics, prostate size and shape, pre- and post-op IPSS and Qmax, and post-void residual. Patient-reported outcomes such as pain during the procedure and satisfaction of the procedure were also collected and analysed as well as complication rates. **Results:** The data of 82 patients undergoing LA Rezum in the above time period were collected and analysed. They had a mean prostate size of 53cc (minimum 21cc and maximum of 100cc). The results showed significant improvement in voiding parameters, with Qmax improving by 40.1% (*p* < 0.05) and PVR by 40.8% (*p* < 0.05). Patients similarly reported improved symptoms, with IPSS improving by 54.7% (*p* < 0.05) and QOL scores by 54.1% (*p* < 0.05). The procedure had a high degree of satisfaction, with 36 of the 49 patients who completed the post-procedure questionnaire recording an overall satisfaction of 9 or 10 out of 10. The mean intraoperative visual analogue (VAS) pain score was 3.5. **Conclusions:** Rezum is a minimally invasive procedure that has been seen to produce significant and reliable improvements in patients’ lower urinary tract symptoms and voiding dynamics. It has a low complication rate, is tolerated well and is readily performed under local anaesthetic in the ambulatory setting.

## 1. Introduction

Currently, in the UK, it is estimated that 1 in 3 men will suffer from lower urinary tract symptoms (LUTSs) secondary to benign prostatic hyperplasia (BPH) at some point in their life, and 1 in 10 will require surgery for it [1]. While transurethral resection of the prostate (TURP) has traditionally been the gold standard for treating symptoms of BPH, it has been associated with relative drawbacks—notably post-operative bleeding, infection and requirement for an inpatient stay.

Novel cavitational procedures such as ‘Holmium laser enucleation of the prostate’ (HOLEP) and ‘Green light laser prostatectomy’ (GLLP) offer alternatives with reduced bleeding risk; however, they still require general anaesthetic. Recent minimally invasive surgical therapies, commonly referred to as MISTs, developed for BPH include Rezum, iTind and Urolift, and they offer an alternative for more frail patients or those with smaller prostates who do not want a more invasive procedure [2].

Rezum therapy was developed in 2015 and involves the injection of heated water vapour into the prostate, causing local coagulative necrosis [3]. It can be done either under general or local anaesthetic, with the local anaesthetic approach having the benefit of being suitable for more frail patients or those who would prefer an ambulatory procedure. Compared to other MIST procedures, Rezum also has the advantage of being ejaculatory sparing and being suitable for patients with a large median lobe. Furthermore it has been shown to be effective both in terms of significantly improving urinary flow parameters such as Qmax and post-void residual (PVR) and patient-reported outcomes such as IPSS [4].

However, despite a significant advantage of Rezum being that the procedure can be performed in a clinic setting under local anaesthetic, much of the current data focuses on the patient undergoing the procedure under a general anaesthetic and there is relatively limited evidence for the local anaesthetic approach. In our institution, patients with LUTSs who prefer an ejaculatory sparing procedure or who are unfit for general anaesthetic are being offered Rezum as a day-case procedure under local anaesthetic [5]. We therefore aimed to analyse the patients undergoing local anaesthetic Rezum in our institution to establish its tolerability, effectiveness and complication rate.

## 2. Materials and Methods

The case notes of all patients who underwent the Rezum procedure under local anaesthetic between May 2023 and September 2025 within our unit were retrospectively analysed. We recorded patient demographics including age, prostate size, and preoperative medications.

### 2.1. Patient Selection

Patients were offered Rezum therapy after being reviewed in a urology one-stop clinic. Patients who had LUTSs, evidence of bladder outflow obstruction on urinary flow studies, and whose symptoms were refractory to medical management were offered Rezum therapy. Patients were counselled on alternative therapies, including, where appropriate, Holmium laser enucleation of the prostate (HoLEP), green light laser prostatectomy (GLLP), and other MIST therapies including iTind and Uroloift. Patients with larger prostates (>80cc) were not offered Rezum therapy unless they were considered unfit for a general anaesthetic. Patients who chose Rezum therapy after appropriate counselling were then added to a waiting list. Patients who underwent the procedure under general anaesthetic were excluded from this study.

### 2.2. Rezum Procedure

The Rezum procedure was done in an outpatient clinic setting with the support of specialist urology nursing staff. The procedure was primarily carried out by one of three consultants. Local anaesthetic was given via a transperineal ultrasound-guided periprostatic block and urethral lidocaine gel. Lidocaine gel (2 × 11 mL) was inserted into the urethra. A penile clamp was applied and kept there for 10 min. The patient was then placed in the lithotomy position and the ultrasound probe was inserted rectally. The lateral margins of the prostate were identified and the perineal skin overlying this was marked on either side. The area was then sterilised with cholorprep and 5 mL 1% lidocaine, with 1:200,000 adrenaline injected at marked points. A larger access needle was passed at the marked points, a spinal needle was passed through and 10 mL 1% lidocaine was injected through this, anaesthetising the neurovascular bundle on each lateral aspect of the prostate. Ultrasound was used to ensure the anaesthetic reached the correct plane.

The Rezum device with a cystoscope was then inserted into the urethra. The patient’s prostatic anatomy was visualised and used to determine the number and position of treatments required. A needle was inserted into the prostatic tissue and a vapour activation button was held to deliver the required treatment. The number of Rezum treatments each patient received was left to the individual surgeon’s discretion in line with the manufacturer’s recommendations. The patient was monitored throughout and could request that the procedure be stopped if it was not tolerable. A urethral catheter was inserted with a plan to be removed in 7–10 days.

The patient was given a 5-day course of ciprofloxacin prophylactically as per local policy and following advice from the trust’s microbiologist (given the potential side effects of fluroquinolones, patients were counselled with regard to side effects).

After the procedure they were asked to fill out a ‘visual analogue pain score’ from 1 to 5, and a ‘patient satisfaction score’ from 1 to 10.

### 2.3. Follow-Up

All patients were followed up at 3 months routinely to assess symptom improvement and any possible complications. Where patients did not attend initial follow-up appointments, a further appointment was offered. At this appointment patients were asked to record the International Prostate Symptom Score (IPSS) and IPSS quality of life (QOL) score, as well as the Internation Index of Ejaculatory Function (IIEF). They then underwent flow studies, and Qmax and PVR was assessed. They were then reviewed in clinic by a specialist BPH nurse and any ongoing symptoms or complications were reviewed.

### 2.4. Endpoints

Primary outcomes were changes in Qmax, post-void residual (PVR), International Prostate Symptom Score (IPSS), and IPSS quality of life score (QOL). This difference was calculated between the patient’s baseline values when they were booked for the procedure and at 3 months follow-up. Secondary outcomes were the patient’s satisfaction with the procedure as noted immediately post-procedure on a scale of 1 (low satisfaction) to 10 (high satisfaction). Patients perceived pain score as recorded on the visual analogue scale (VAS) from 1 (no pain) to 5 (severe pain). 

### 2.5. Statistics

Data were analysed using ‘Microsoft Excel’ and ‘R: R Project for Statistical Computing v4.3.2’ [6]. Normality was tested visually and using the Shapiro–Wilk test. Nonparametric data were analysed with the Wilcoxon signed rank test and parametric data with the paired *t*-test. Mean values were reported with their standard deviation. A *p* value of <0.05 was treated as significant.

### 2.6. Ethical Considerations

This study was done as an audit and, in accordance with the NHS Health Research Authority, did not require formal ethical approval.

## 3. Results

A total of 82 patients underwent a Rezum procedure under local anaesthetic within our department in the above time period. Of these, 75 (90%) attended follow-up at 3 months. The mean patient age was 71.0 (+/−9.05) and prostate size was 53.0cc (+/−15.96). Five (6.1%) patients had prostates above 80cc and the largest prostate in the patient group was 100cc. In the cohort, 28 (34.1%) patients were noted to have a large median lobe and 11 (13.4%) a high bladder neck. The mean number of injections delivered was 4.1 (+/−1.62). In the patient sample, all patients but one tolerated the procedure well, with that individual asking for it to be stopped after two treatments were delivered. The mean PSA pre-procedure was 4.2 and post-procedure was 4.1.

### 3.1. QMAX and PVR

**Figure 1:**
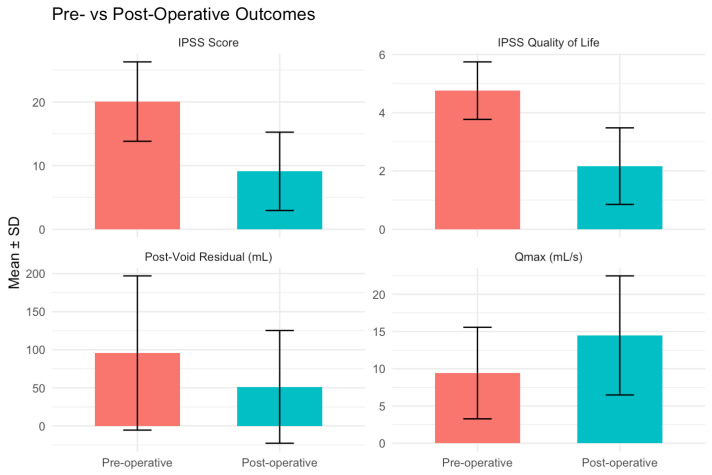
Pre and post operative outcomes.

Patients were found to have a significant reduction in PVR (*p* =< 0.05) and improvement in QMax (*p* =< 0.05). Median pre- and post-op values are shown in Table 1. Individual improvement in patient Qmax and PVR can be seen in Figure 1.

### 3.2. IPSS and IPSS QOL

The patient-reported IPSS improved from pre- to post-procedure by 54.7% (*p* =< 0.05) and QOL by 54.1% (*p* =< 0.05) with the mean averages shown below (Table 1).

### 3.3. Tolerability

**Figure 2:**
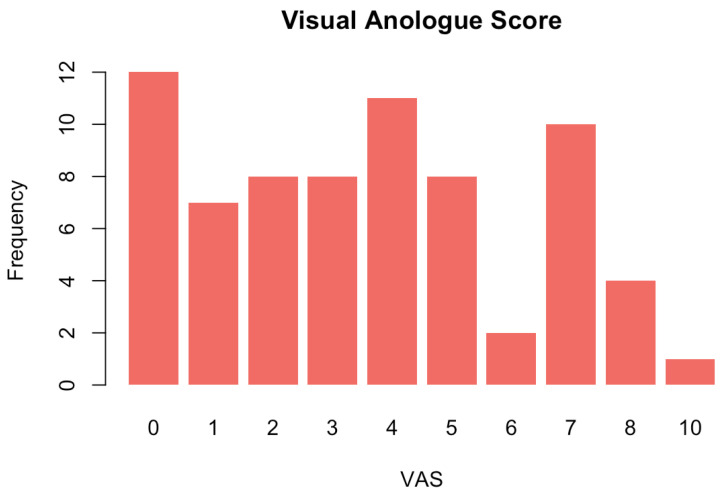
Frequency of Visual Anologue Scores.

All but one patient tolerated the procedure. One patient asked for the procedure to be stopped after two injections were given. One patient had to be admitted post-procedure for pain relief. Of the 49 (59.8%) patients that completed the post-procedure satisfaction questionnaire, 36 (73%) patients recorded a score of 9 or 10 out of 10. The intraoperative visual analogue score (VAS) was recorded in 71 (86%); patients had a mean score of 3.6. The frequency of recorded VASs can be seen in Figure 2.

### 3.4. IIEF

The IIEF had a poor response rate, with only four patients filling it out preoperatively and 27 patients post-operatively, and only three patients filling it out both pre- and post-operatively. There was a mean score of 63 preoperatively and 47 post-operatively. While it could not be considered valid to analyse this given the poor response rate—it can be anecdotally noted that no patient reported new ejaculatory disfunction when asked about this at the follow-up clinic.

### 3.5. LUTS Medications

A total of 67 patients had been treated preoperatively with medications to improve bladder outflow symptoms. This included 56 with alpha blockers and 28 with 5-alpha reductase inhibitors. This reduced to three patients requiring medical therapy with alpha blockers post-procedure.

### 3.6. Complications

No patients had a Claven–Dindo complication ≥ III. Ongoing pain relief requirement meant that one patient required admission post-procedure. It was noted that five patients had an ongoing catheter requirement after the procedure; however, all five of these had a catheter pre-procedure. Two of these patients had recurrent UTIs thought to be due to poor bladder emptying. Furthermore, two patients were unsatisfied with the symptomatic improvement after the procedure and were re-listed for a green light laser prostatectomy (GLLP). One patient experienced tendinosis due to ciprofloxacin. Of the five patients with a prostate size above 80cc, one patient required re-listing for GLLP while the rest had adequate symptom improvement and a successful trial without a catheter. A full table listing the complications is shown below (Table 2).

## 4. Discussion

In this retrospective case series of 82 patients undergoing Rezūm therapy under local anaesthetic, it can be demonstrated that the procedure is feasible, well tolerated, and associated with improvements in both objective voiding parameters and patient-reported outcomes. At three months follow-up, patients experienced significant improvements in Qmax and post-void residual volume, alongside substantial improvement in IPSS and quality of life scores. The procedure was associated with high patient satisfaction, moderate intraoperative pain scores, and a low complication rate.

Improvements in voiding dynamics and symptoms were in keeping with the previous literature. A systematic review by Gemma et al. (2024) found that Qmax improved by 53.5% on average, ranging from 39% to 87%, comparable to our finding of a 40.1% improvement [7]. Similarly, our result of a decrease in the IPSS of 54.1% falls in the range seen in the same review (47–53%). Importantly, McVary et al. demonstrated that among 136 patients undergoing Rezum, these improvements were durable over three years, suggesting that the symptomatic benefits observed in this cohort are likely to be sustained [8]. All the studies in the above systematic review included a mix of general-anaesthetic and local-anaesthetic Rezum patients. Our comparable results, when performed exclusively under local anaesthetic, serve to emphasise the effectiveness of the local anaesthetic technique.

Performing Rezum under local anaesthetic in a way which is safe and tolerable and results in good outcomes has important clinical implications. It allows older, more frail patients to undergo treatment for bladder outlet obstruction when they may not be fit for cavitational procedures such as HoLEP, green light laser prostatectomy or TURP, and also offers a solution for those patients who are motivated and keen to preserve their ejaculatory function without the need to undergo a general anaesthetic procedure. It can also have implications with regard to service management—allowing treatment to be delivered in an outpatient setting and reducing the reliance on operative theatre capacity.

Although in this unit a transperineal periprostatic block with urethral lidocaine gel is opted for, there exist alternative local anaesthetic protocols. A recent review by Wirtzfeld et al. found that the main alternative method involved transurethral intraprostatic anaesthesia with a Schelin catheter. This involves inserting the catheter with a retractable needle and directly inserting local anaesthetic clockwise into the prostate. The review found no significant difference in patient-reported pain scores between the periprostatic block and transurethral intraprostatic anaesthesia and indeed found both resulted in a tolerable procedure [9].

It is a notable point that four out of five patients with larger prostates (>80cc) have experienced symptomatic improvement from the procedure. This again fits with current evidence. An observational study by Garden et al. compared outcomes for 36 patients with larger prostates (>80cc) to those with smaller prostates (<80cc) [10]. They found that those with larger prostates experienced similar improvements in voiding dynamics and patient-reported symptom scores as those with smaller prostates; furthermore, they showed that Rezum allowed about 30% of these patients to stop treatment with alpha blockers. However, they did note that these patients had a slightly longer time to successful TWOC. A potential area of future research could focus on the role that LA Rezum could play for these patients with large prostates (>80cc) who are unsuitable for more definitive procedures.

One patient in the above study was thought to have developed tendinosis secondary to ciprofloxacin use. Antibiotic choice was in conjunction with local guidance and in liaison with microbiological colleagues. In the above cohort, on the balance of risks, it was deemed appropriate to discharge patients on a short course of fluroquinolones with relevant counselling. Given the increasing regulatory concern around fluroquinolones, antibiotic choice is regularly reviewed and would change if the advice changes.

This study has several limitations. Its retrospective design introduces the potential for selection and information bias, and the absence of a control group limits direct comparison with alternative treatments. Follow-up was limited to three months, restricting assessment of long-term durability; however, previous randomised data suggest that maximal improvement following Rezūm occurs by three months and is subsequently maintained. Although follow-up rates were high, a small proportion of patients did not attend follow-up and could not be included in the analysis. As noted, IIEF response rates were low and this limits the validity of these results. Finally, an intention-to-treat analysis was not possible, which may bias the reported outcomes.

## 5. Conclusions

The Rezum procedure can feasibly and safely be carried out under local anaesthetic and is well tolerated by patients with a low complication rate. The procedure resulted in quantitative improvements in patients’ Qmax and PVR at 3 months. Patient-reported outcomes show a high degree of satisfaction, with the majority of patients reporting improved symptoms and QOL post-procedure.

## Figures and Tables

**Table 1:** Pre and post operative outcomes.

	Pre-Procedure	Post-Procedure (~3 Months)	*p*-Value
Qmax	9.45 (5–11.7)	13.3 (9.7–17.4)	<0.05
PVR	49 (23.25–140)	29 (9.5–61)	<0.05
IPSS	20.1 (+/−6.24)	9.1 (+/−6.15)	<0.05
QOL score	4.8 (+/−0.99)	2.2 (+/−1.31)	<0.05

**Table 2:** Complications.

Complication	Age	Prostate Volume	Management	Outcome
Ongoing catheter requirement	83	NA	Failed TWOC on two occasions. Patient not keen for repeat or alternative procedure at the current time	Patient managing with ongoing catheter
Ongoing catheter requirement and recurrent UTIs	75	72cc	Failed TWOC	Relisted for GLLP—awaiting procedure
Ongoing catheter requirement and recurrent UTIs	68	72cc	Ongoing ISC use	Planned for further urodynamic studies to plan further treatment
Ongoing catheter requirement	75	58cc	Failed TWOC	Patient keen for ongoing ISC use, does not want further treatment
Ongoing catheter requirement	70	50cc	Flowtrometery indicated poor bladder emptying so initially continued with ISC	Repeat flowtrometery 6 months post-procedure which indicated good bladder emptying. Able to stop ISC.
Ongoing LUTSs	66	50cc	Relisted for GLLP	GLLP
Ongoing LUTSs	50	46cc	Relisted for GLLP	GLLP
Tendinosis (possibly due to ciprofloxacin)			Pain resolved after antibiotic course complete	Self-resolved
Pain post-procedure	84	40cc	Admitted for pain relief overnight. Pain self-resolved in 24 h	Discharged after 24 h

NA—not available.

## Data Availability

The data presented in this study are available on reasonable request from the corresponding author. The data are not publicly available due to restrictions related to patient confidentiality.
